# A new Sky Island species of *Vaejovis* C. L. Koch, 1836 from Sonora, Mexico (Scorpiones, Vaejovidae)

**DOI:** 10.3897/zookeys.760.22714

**Published:** 2018-05-28

**Authors:** Diego A. Barrales-Alcalá, Oscar F. Francke, Tom R. Van Devender, Gerardo A. Contreras-Félix

**Affiliations:** 1 Posgrado en Ciencias Biológicas, Universidad Nacional Autónoma de México (UNAM), Avenida Universidad 3000, Ciudad Universitaria, Coyoacán, Ciudad de México, México; 2 Colección Nacional de Arácnidos (CNAN), UNAM, 3er circuito exterior S/N, Ciudad Universitaria Coyoacán, Ciudad de México, México, CP 04310; 3 Greatergood.org, 6262 N. Swan Road, Suite 150. Tucson, Arizona 85718, USA

**Keywords:** Diversity, pine-oak forests, scorpions, Speciation

## Abstract

*Vaejovis islaserrano* **sp. n.** is described from the Sierras Elenita and La Mariquita, Municipio de Cananea, Sonora, Mexico. This species belongs to the “*vorhiesi*” group of the genus *Vaejovis* and inhabits pine-oak forests in northern Mexico. This species is compared to its most similar species. This new species presents an interesting morphological difference from the rest of the species in the species-group: the absence of a subaculear tubercle or spine.

## Abstract (es)

Se describe *Vaejovis islaserrano* **sp. n.** de las Sierras Elenita y La Mariquita, en el Municipio de Cananea, Sonora, México. Esta especie pertenece al grupo “*vorhiesi*” dentro del género *Vaejovis* y que habita en los bosques de pino y encino del norte de México. Se le compara con las especies más similares morfológicamente. Esta nueva especie presenta una característica morfológica interesante para las especies del grupo: la ausencia de un tubérculo o espina subaculear.

## Introduction

Approximately 2300 species of scorpions have been described worldwide (Santibáñez-López et al. 2016). The family Vaejovidae is the most diverse scorpion family on North America, with at least 211 described species. The most recent works on Vaejovidae focused mainly on the subfamily Syntropinae (González-Santillán and Prendini 2013, 2014, 2015). In addition, seven new species in the genus *Vaejovis* C. L. Koch, 1836 within the “*vorhiesi*” complex have been described from the Sky Island mountain ranges in the southwestern United States and northwestern Mexico (Graham et al. 2012; Sissom et al. 2012; Ayrey 2012, 2013, Ayrey and Webber 2013, Ayrey and Soleglad 2014, 2015); six of the new species are from the United States and only one from Sonora, Mexico.

The Madrean Archipelago (Bezy and Cole 2014, Bezy et al. 2017) located between the northern Sierra Madre Occidental in Sonora, Mexico and the Mogollon Rim in central Arizona contains 55 Sky Islands and Sky Island complexes (mountain ranges connected by oak woodland passages), 32 of them in Sonora (Van Devender et al. 2013). Sky Islands are isolated mountain ranges with crowns of oak woodland and pine-oak forest surrounded by lowland ‘seas’ of thorn scrub, desert scrub, or desert grassland. The Sky Islands are well known for their high biodiversity (Van Devender et al. 2013); the fauna and flora of the upper ranges are isolated from each other, and there are endemic species in some groups, but the rates of endemism in the Sky Islands are low compared to oceanic islands (Bowers and McLaughlin 1996, Reina-Guerrero and Van Devender 2005), or the Sierra Madre Occidental near Yécora, Sonora (Van Devender and Reina-Guerrero 2016). The *Vaejovis* species distributed in the area are the exception, reflecting an important evolutionary radiation on Sky Island mountain tops. Bryson et al. (2013) suggested that each isolated mountain range could harbour an endemic scorpion of the “*vorhiesi*” species group. If this is true, several species still await discovery and description from the Madrean Archipelago.

The Sierra La Mariquita and La Elenita are two interconnected Sky Islands in northern Sonora located northwest of the copper mining town of Cananea, only 26 km south of the Arizona border. The Sierra La Mariquita and Sierra Elenita are connected by oak woodland in Puerto Cananea. They are geographical sister ranges to the Huachuca Mountains of Arizona.

On the southwestern side, the Sierras La Mariquita rise from 1230 to 2498 m and La Elenita from 1230 to 2305 m at the summits. The vegetation varies from desert grassland and oak woodland on the lower slopes to pine-oak forest above. The montane forest is dominated by Apache pine (*Pinus engelmannii*) and southwestern white pine (piñón, *Pinus strobiformis*). The Sierra Elenita has similar vegetation but the pine-oak forest is better developed in a large area. Both ranges are in the San Pedro River drainage, which flows northward into Arizona.

The Sierra La Mariquita was visited in June 2009, September 2010, and August 2013 as part of the Madrean Archipelago Biodiversity Assessment (MABA) program of the Sky Island Alliance. The Sierra Elenita was visited in September 2016 as part of the Madrean Discovery Expedition program of GreaterGood.org. Animal and plant observations and collections from these and other Sonoran Sky Islands are available in the Madrean Discovery Expedition (MDE) database (madreandiscovery.org; linked to the MABA database). Here we describe *Vaejovis islaserrano* sp. n. in the “*vorhiesi*” complex collected during these expeditions, as an addition to the scorpion biodiversity of the Sky Island Region.

## Materials and methods

Nomenclature and mensuration follows Stahnke (1970), except for trichobothrial terminology after Vachon (1974), cheliceral dentition after Vachon (1963); metasomal, pedipalpal carination, as well as the hemispermatophore terminology after González-Santillán and Prendini (2013); metasomal setae counts, modified from Santibáñez-López and Sissom (2010); telotarsal spination and setal counts following Contreras-Félix et al. (2015); terminology for the lateral eyes follows Loria and Prendini (2014); laterobasal aculear serrations (= LAS) terminology follows Fet et al. (2006); hemispermatophores were dissected following Vachon (1952) , and cleared using the technique of Álvarez-Padilla and Hormiga (2008). Higher level taxonomy of scorpions follows Sharma et al. (2015) and Prendini and Wheeler (2005). Finally, photographs of each metasomal segment using UV light were taken following the recommendations of Prendini (2003) and Volschenk (2005). Images were taken using a digital camera Leica DFC490 (8 mp) attached to a Leica Z16 APO A microscope and prepared using “Leica Application Suite-version 4.3.0 (Build: 600)”. Pictures were edited with software Adobe Photoshop CS6. The map was generated with ESRI ArcGIS online suite. Finally, the measurements were taken with an ocular micrometre calibrated at 10X, and are given in millimetres.

## Systematics

### Family Vaejovidae Thorell, 1879

#### Genus *Vaejovis* C. L. Koch, 1836

##### Vaejovis islaserrano sp. n.

Taxon classificationAnimaliaScorpionesVaejovidae

http://zoobank.org/998D60A1-C6C2-4D2F-81BF-361B33CD3221

Figs 1 , 2 , 3 , 4 , 5 , 6 , 7 , 8 , 9

###### Type material.

Holotype Male, MEXICO: Sonora, Municipio Cananea, vicinity of Observatorio Astrofísico Guillermo Haro, Sierra La Mariquita (31.05444°N, 110.38244°W, 2422 m elev) 03-VIII-2013. Cols: T. R. Van Devender, J. D. Palting, and G. Molina. 1 ♂ (CNAN-T01207).

Paratypes: Same data as the holotype 4 males and 5 females (CNAN-T-01208); 2 males and 2 females (AMNH). MEXICO: Sonora, Cananea, Sierra La Elenita. Near “El 15” (31.00252°N, 110.38944°W, 1911 m) 30-IV-2016. Cols: D. Barrales, J. Cirett, I. Ochoa. Pine-Oak forest.

###### Etymology.

The specific epithet is regarding the distribution of the species in the highlands of the Sonoran desert and it is composed by the words in Spanish “isla” in reference of island and “sierra” as in mountain range, being the adjective “serrano” and together they compose the name islaserrano, which is used as a noun in apposition.

###### Diagnosis.

*Vaejovis islaserrano* sp. n. belongs to the “*vorhiesi*” group due to the presence of the following characters: the presence of a sclerotized mating plug in the spermatophore; trichobothria *ib* – *it* on the base of the fixed finger of the pedipalp chela; the absence of setae on the prolateral and retrolateral sides on the first pair of legs. This is a relatively small scorpion, with adult total length ranging from 18 mm to 24 mm (Table 1). Sternite V with a noticeable whitish oval spot on the posterior fifth, also present on sternite VII. Vesicle of the telson, elongated more than twice longer than wide (L/W: 2.44), and thin, almost as wide as deep (W/D: 1.12). LAS present on both sides of the aculeus. Pedipalp chela fingers dentate margins straight, without scalloping.

**Table 1.** Measurements on selected specimens of *Vaejovis islaserrano* sp. n. The measurements are given in mm.

	Holotype ♂	Paratype ♂	Paratype ♂	Paratype ♂	Paratype ♂
Total L	19.5	20.3	18.3	18.3	20
Carapace L	2.5	2.7	2.4	2.1	2.7
Carapace W	1.3	1.4	1.3	1.3	1.4
Mesosoma L	5.4	5.9	5.5	5.4	6.1
SMI L/W/D	1.2/1.5/1.2	1.3/1.5/1.2	1.1/1.4/1.1	1.2/1.5/1.2	1.21.5/1.3
SM II L	1.5	1.5	1.3	1.4	1.4
SM III L	1.6	1.6	1.5	1.5	1.5
SM IV L	2.1	2.1	1.9	1.9	2
SM V L/W/D	3/1.3/1.1	31.2/1.1	2.6/1.3/1	2.71.2/1	2.9/1.3/1.2
Metasoma L	9.4	9.5	8.4	8.7	9
Vesicle L/W/D	2.2/0.9/0.8	2.2/0.9/0.8	2/0.9/0.7	2.1/0.8/0.6	2.2/1/0.8
Femur L/W/D	2.1/0.6/0.5	2.3/0.7/0.5	2.1/0.7/0.4	2.2/0.6/0.4	2.4/0.7/0.4
Patella L/W/D	2.4/0.8/0.7	2.5/0.8/0.6	2.3/0.8/0.5	2.4/0.7/0.6	2.7/0.8/0.6
Chela L/W/D	2/0.8/0.8	2/0.8/0.8	1.7/0.7/0.8	1.8/0.8/0.8	2/0.8/0.8
Movable finger L	2.3	2.3	2	2.2	2.5
Fixed finger L	1.8	1.8	1.7	1.8	2.1
Pectinal tooth counts	13–14	14–14	13–13	14–15	14–15
	**Paratype** ♀	**Paratype** ♀	**Paratype** ♀	**Paratype** ♀	**Paratype** ♀
Total L	20.3	23.8	24.1	23.1	23.9
Carapace L	3.2	3.3	3.4	3.3	3.4
Carapace W	1.7	1.8	1.9	1.8	1.9
Mesosoma L	7.4	7.8	7.9	7.4	7.6
SMI L/W/D	1/1.4/1.1	1.4/1.8/1.5	1.4/1.9/1.5	1.3/1.6/1.4	1.4/2/1.5
SM II L	1.3	1.6	1.5	1.6	1.6
SM III L	1.4	1.7	1.9	1.7	1.7
SM IV L	1.6	2.1	2.3	2.2	2.3
SM V L/W/D	2.4/1.2/1	3.31.6/1.3	2.91.6/1.4	3.21.6/1.3	3.3/1.6/1.4
Metasoma L	7.7	10.1	10	10	10.3
Vesicle L/W/D	2/0.9/0.7	2.6/1/1.1	2.8/1.2/1	2.4/1.1/0.9	2.6/1.2/0.9
Femur L/W/D	2.1/0.7/0.4	2.80.9/0.5	3/1/0.6	2.8/0.9/0.6	3/1/0.7
Patella L/W/D	2.4/0.7/0.6	3.1/1/0.8	3.2/1/0.8	3.2/1/0.8	3.4/1.1/0.9
Chela L/W/D	1.8/0.8/0.8	2.3/1/1	2.5/1.1/1.1	2.3/1.1/1.1	2.5/1.1/1.1
Movable finger L	2.1	3	3.1	2.9	3.2
Fixed finger L	1.9	2.5	2.5	2.3	2.6
Pectinal tooth counts	12–13	13–14	12–12	12–12	13–13

*Vaejovis islaserrano* sp. n. is most similar to *Vaejovis bandido* Graham, Ayrey & Bryson, 2012, from Sierra Los Ajos, Sonora, but it is easily differentiated by the following characters: the presence of a subaculear spine in *V. bandido*, whereas in *V. islaserrano* sp. n. does present a vestigial subaculear spine: the presence of a caudal gland of the telson evident on adult males of *V. islaserrano* sp. n., whereas in *V. bandido* it is not evident; the hemispermathophore presents an apical crest on the lamella in *V. bandido*, whereas *V. islaserrano* sp. n., presents a lamella without crest. Another species closely related to *V. islaserrano* sp. n. is *V. vorhiesi* Stahnke, 1940, from the nearby Huachuca Mountains, Arizona, that can be differentiated as follows: *V. vorhiesi* presents a subaculear spine, whereas *V. islaserrano* sp. n. does not present a subaculear spine. Finally, *Vaejovis cashi* Graham, 2007, from the Chiricahua Mountain in Arizona, differs from *V. islaserrano* sp. n. in the following characters: smaller size (19 to 22 mm); a small aculear spine present in *V. cashi*, absent in *V. islaserrano* sp. n.; the hind laminar hook on the hemispermathophore weakly developed, almost fused with the other hook in *V. islaserrano* sp. n. versus hooks well differentiated with a deep depression between them in *V. cashi*.

###### Description of the holotype male

**Figure 1.**
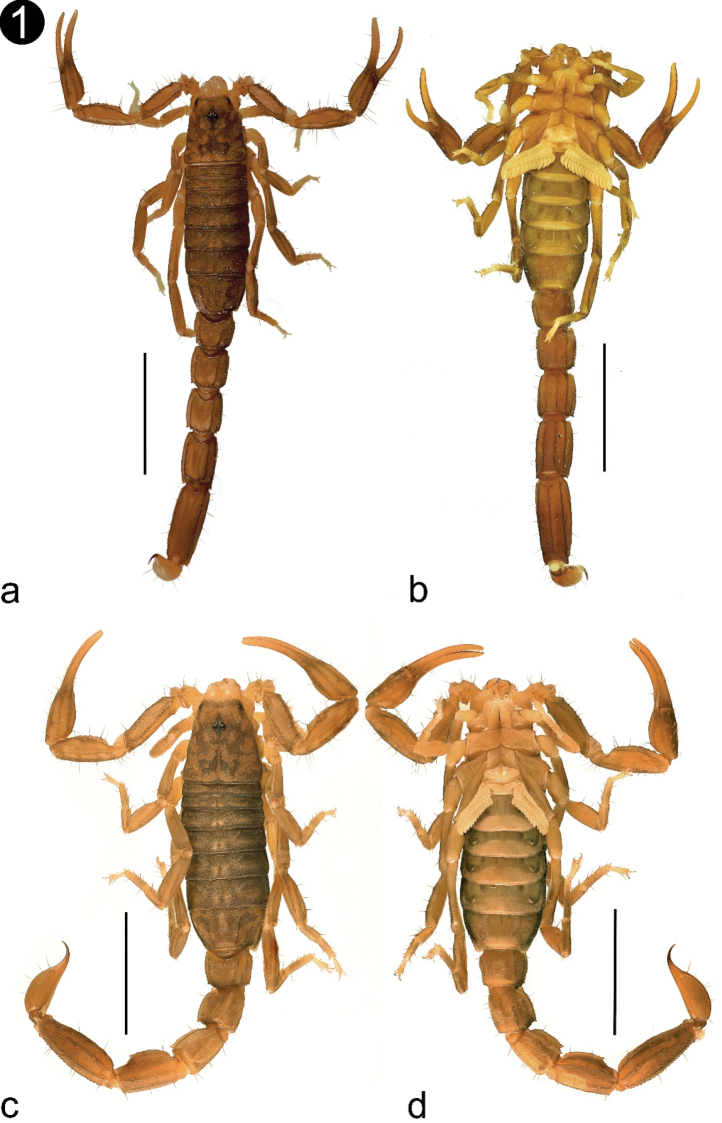
Habitus of *Vaejovis islaserrano* sp. n. **a, b** Habitus of the Holotype male **c, d** Paratype female **a, c** dorsal view **b, d** ventral view. Scale bars: 5 mm.

(Fig. 1a, b). ***Coloration***: Chelicerae, pale yellow coloration, with a black pattern on distal margin of chelae. Carapace, pale yellow coloration, with a diffuse fusco-piceus pattern. Mesososma, tergites pale yellow, with a diffuse fusco-piceus pattern. Sternites III-VII pale yellow, with a diffuse dark pattern on the sides; sternite V with a noticeable pale oval spot on posterior fifth, and sternite VII presents also a noticeable triangular spot on the posterior fourth. Metasoma, segments I-V pale yellow, with a very diffuse black pattern, more evident dorsally on each segment. Telson, pale yellow coloration with a diffuse fusco-piceus pattern present on the ventral face and additionally on the dorsal face, but faint. Pedipalp, Femur, patella and chela pale yellow with a diffuse dark coloration intense at the margins of each segment and on the carinae. Legs, pale yellow, with a diffuse dark coloration present, denser on prolateral face of femur and patella and on prolateral and retrolateral margins of basitarsus.

**Figure 2.**
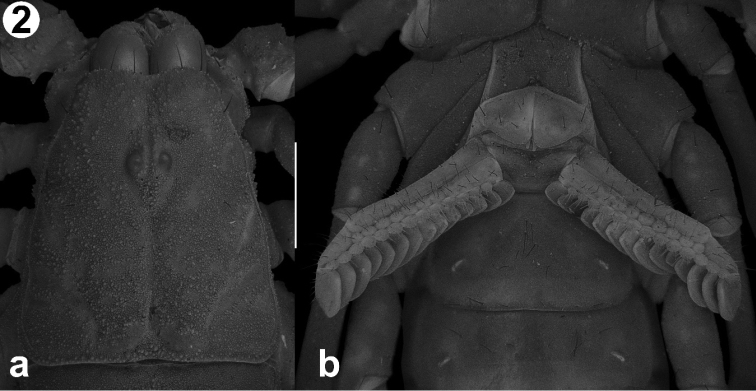
**a** Carapace of the holotype male of *Vaejovis islaserrano* sp. n. dorsal view **b** Holotype mesosoma, ventral view, showing the pectinal teeth and sternite. Scale bar: 1 mm.

Prosoma. *Chelicerae*: Serrula present, well-developed. Dorsal margin of movable finger with the basal denticle smaller than the median followed by two small subdistal denticles and a larger distal denticle; ventral edge of movable finger smooth. Fixed finger with basal denticle bicuspid, subdistal denticle small and distal denticle larger compared to each other. *Carapace* (Fig. 2a): Anterior margin slightly concave, almost straight; anteromedian longitudinal sulcus shallow; surface of carapace minutely granular on area surrounding the median ocelli, rest of surface granular. Ocular tubercle with superciliary carinae lower than medial ocelli; lateral ocelli type 3A (Loria and Prendini 2014).

*Mesosoma*: Tergites I-II, shagreened, with a granular pattern confined to posterior margin; tergites III-VI with anterior half shagreened and posterior half noticeably granular, with median carina present on posterior half of each segment (Fig. 2b). Tergite VII with strongly developed submedian and lateral carinae, paramedian carinae reaching posterior margin; intercarinal surface noticeably granular. Sternites III-VI smooth, slightly granulated on posterolateral margins; sternite VII intercarinal surface shagreened, slightly granular on the sides and with 11 setae; lateral carinae strong, composed by a row of aggregated granules. Pectinal tooth count: 13–14 (Fig. 2b).

**Figure 3.**
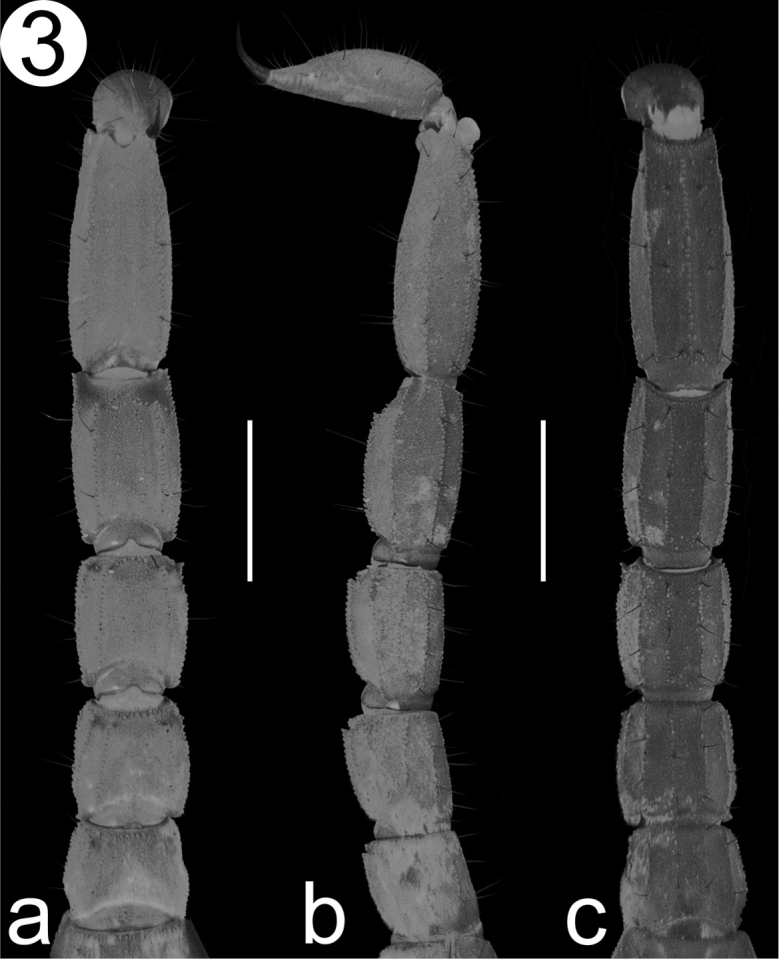
Detail of the metasoma, from the Holotype male. **a** Dorsal view **b** Lateral view **c** Ventral view. Scale bar: 2 mm.

*Metasoma* (Fig. 3a–c): Dorsal lateral and lateral median carinae on segments I–IV strong, composed by a single line of granules and the distalmost slightly larger than the preceding (Fig. 3a); lateral inframedian carinae on segments I–III strong, composed by a single row of granules and present along the entire segment, on segment IV vestigial, composed by small scattered granules on distal half (Fig. 3b); ventral lateral carinae on segments I–IV strong, composed by a single row of granules; ventral submedian carinae on segment I weak, composed by a row of low granules just above the surface, on segments II–IV, strong, composed by a single row of raised granules. Dorsal and lateral intercarinal surfaces minutely granular, and on ventral face shagreened (Fig. 3c). Segment V: Dorsal lateral carinae strong, composed by a single row of granules on anterior half, wider with scattered granules on posterior half; lateral median carinae strong, composed by an irregular row of granules and present on basal two thirds; ventral lateral carinae strong, composed by a single row of granules; ventral median carina strong, composed by a single row of granules and not reaching posterior margin. Setae count on metasomal segments I–IV as follows: DL: 0/0/1/2; LM: 1/1/0/3; LI: 1/1/0/3; VL: 2/2/0/3; VS: 2/2/0/3. On segment V: DL: 3; LM: 2-3; VL: 3; VM: 3 (Full variation of setal counts in the metasoma, is given in Table 2).

**Table 2.** Metasomal setal counts on selected segments of the type series of *Vaejovis islaserrano* sp. n. Abbreviations: DL: Dorsal lateral; LM: Lateral median; LI: Lateral inframedian; VL: Ventral lateral and VS/M; ventral submedian/median carinae.

Metasomal setae counts										
DL	0/1/1/2/3	0/1/1/2/3	0/1/1/2/3	0/1/1/2/3	0/1/1/2/3	1-0/1/1/2/3	1-0/1-0/1/2/3	1-0/1/1/2/3	0/1/1/2/3	0/1/1/2/3
LM	0/1/1/2/3-2	0/1/1/2/2	0/1/1/2/2	0/1/1/2/2	0/1/1/2/2	1/1/2-1/2/3	0/1/1/2/2	0/1/1/2/3	0/1/1/2/2-3	0/1/1/2/2
LI	1/0/0/0	1/0/0/0	1/0/0/0	1/0/0/0	1/0/0/0/	1/0/0/0/	1/0/0/0/	1/0/0/0/	1/0/0/0/	1/0/0/0/
VL	2/3/3/3/3	2/3/3/3/3	2/3/3/3/3	2/2/3/3/3	2/3/3/3/3	2/2-3/3/3/3	2/3/3/3/3	2/3/3/3/3	2/3/3/3/3	2/3/3/3/3
VS/M	3/3/3/3/3	3/3/3/3/3	3/3/3/3/3	3/3/3/3/3	3/3/3/3/3	3-1/3/3/3/3	3/3/3/3/3	3/3/3/3/3	3/3/3/3/3	3/3/3/3/3

**Figure 4.**
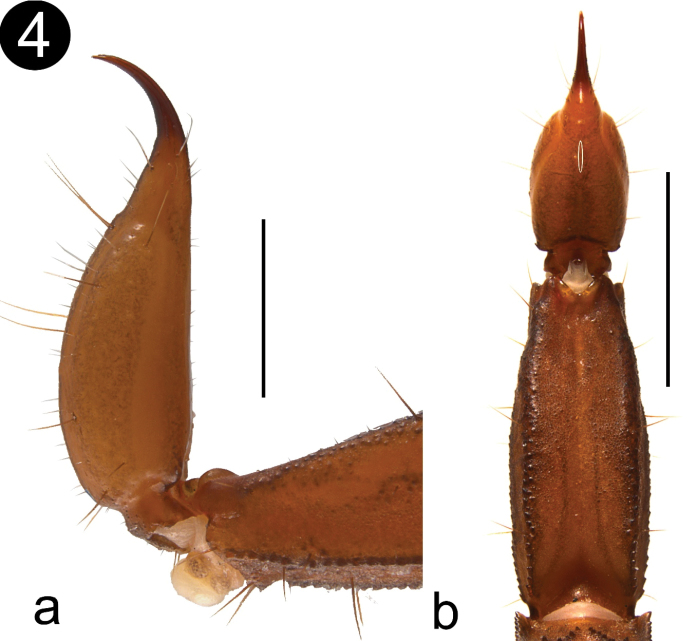
**a** Male telson lateral view, showing the vestigial subaculear tubercle or spine **b** telson dorsal view, showing the faint and elongated shape of the caudal gland, which in this picture is highlighted with a white oval. Scale bars: 1 mm **a**, 5 mm **b**.

*Telson* (Fig. 4): Vesicle elongated, more than twice longer than wide (L/W: 2.44), and thin, almost as wide as deep (W/D: 1.12). Subaculear tubercle vestigial to absent (Fig; 4a) Glandular area on the dorsal face present on distal third, and longer than wide (Fig. 4b). Surface of vesicle smooth on ventral and dorsal faces. LAS present on both sides of the aculeus.

**Figure 5.**
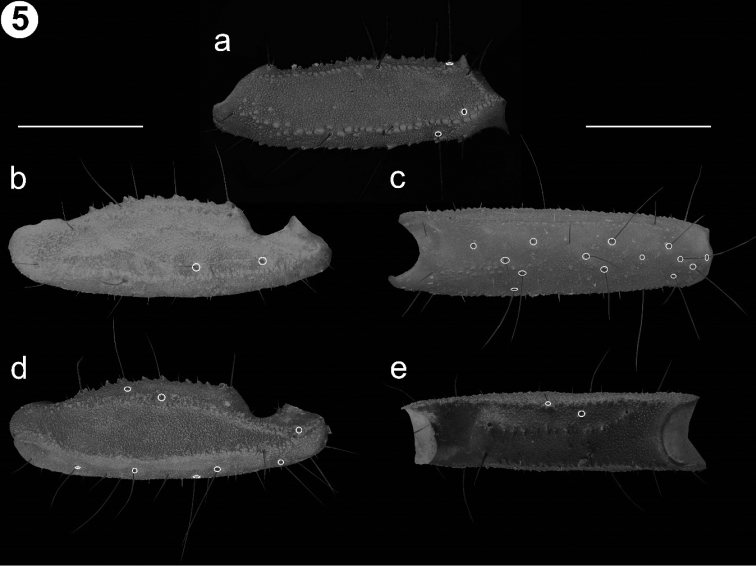
Detail of the segments in the pedipalp of the Holotype male of *V. islaserrano*. **a** Femur, dorsal view **b** Patella, ventral view **c** Patella, retrolateral view **d** Patella, dorsal view **e** Patella, prolateral view. Scale bars: 1 mm.

**Figure 6.**
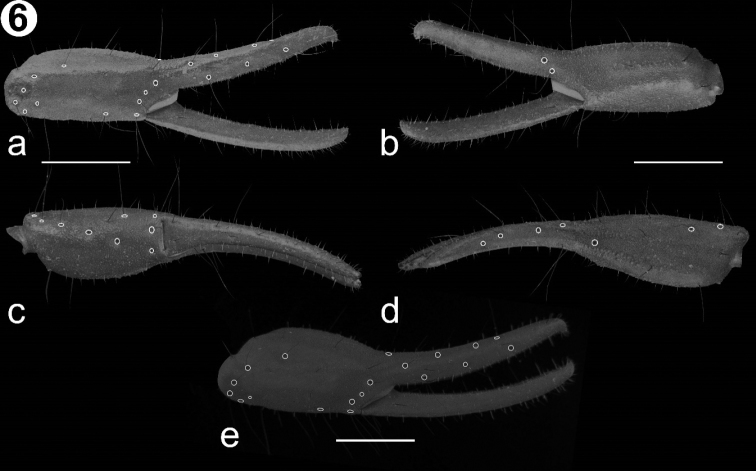
Detail of the chela in *V. islaserrano*. **a** Chela holotype male, retrolateral view **b** Chela holotype, prolateral view **c** Chela holotype, ventral view **d** Chela holotype, dorsal view **e** Chela of the a paratype female of *V. islaserrano* sp. n. retrolateral view. Scale bars: 1 mm.

*Pedipalp* (Fig. 5): Orthobothriotaxic type “C”. *femur* (Fig. 5a) more than three times longer than wide (L/W: 3.5) and slightly wider than deep (W/D: 1.2); dorsal retrolateral and dorsal prolateral carinae strong, composed by an irregular line of granules; prolateral ventrosubmedian carina strong, composed by a line of large granules along the segment; prolateral ventral carina, vestigial, only present by two larger, separate granules; ventral prolateral carina strong, composed by several rows of aggregated granules; ventral median and retrolateral ventral carinae strong, composed by a line of granules; ventral retrosubmedian carina undistinguishable from other granules of the ventral surface; ventral retrolateral carina weak and smooth; retrolateral dorsosubmedian carina strong, composed by an irregular row of larger granules; intercarinal spaces all surfaces are granular. *Patella* (Fig. 5b–e): Three times longer than wide (L/W: 3) and wider than deep (W/D: 1.2). Dorsal prolateral and dorsal retrolateral carinae strong, composed by several rows of granules; prolateral subdorsal carina absent; prolateral median carina strong, composed by a line of scattered large granules; ventral prolateral carina strong, composed by a line of granules; ventral median carina strong, composed by a line of scattered granules and present on more than half of segment; ventral retrolateral carina strong, composed by a line of granules; retrolateral median and retrolateral dorsosubmedian carinae weak, almost absent, composed by scattered small granules and a slight costa. Intercarinal spaces shagreened with some scattered granules on ventral face. *Chela* (Fig. 6a–d): Manus more than twice longer than wide (L/W: 2.5) and as wide as deep (W/D: 1). Dorsal retrolateral carina weak, with a costa and some small granules; retrosubmedian accessory carina weak, composed by several rows of aggregated small granules; dorsal median carina weak, composed by a costa and some small granules; dorsal prosubmedian and dorsal prolateral carinae strong, composed by several rows of aggregated granules; prolateral dorsal, ventral median, ventral prosubmedian, retrolateral subventral accessory and retrolateral dorsal carinae absent; prolateral median and prolateral ventrosubmedian carinae strong, composed by a row of aggregated large granules; ventral prolateral and prolateral ventral carinae vestigial, almost absent, composed by a slight costa and some scattered granules; ventral retrolateral carina faint, almost absent; retrolateral subventral carina weak, only present as costa; retrolateral median carina faint, only differentiated by a small line of granules and a slight costa. Intercarinal surfaces shagreened. Dentate margins of the pedipalp chela fingers straight; fixed finger with five inner accessory denticles, movable finger with six inner accessory denticles.

*Legs*: Telotarsi on legs I-IV with a single line of spinules ventrally and with two distal spinules on each leg (Table 3). Prolateral and retrolateral setae on the telotarsi as follows: 0/0:1/1:1/1:1/1.

**Table 3.** Telotarsi setal counts on selected specimens of the type series of *Vaejovis islaserrano*. Abbreviations: DTS: Distal terminal setae; Pi/Ri: Prolateral internal/Rotrolateral internal.

**Legs Distal Terminal Setae counts**										
DTS	4-4-4-4	2-2-2-2	2-2-2-2	2-3-2-2	2-2-2-2	2-2-2-2	x-2-2-2	2-2-2-2	2-2-x-2	2-2-2-2
	4-4-4-4	2-2-x-2	2-2-2-2	2-2-2-2	2-2-2-2	2-2-2-2	2-4-2-2	2-2-2-x	2-2-x-x	2-2-2-2
**Prolateral and retrolateral setae counts**										
Pi/ri	0/0:1/1:1/1:1/1	0/0:1/1:1/1:1/1	0/0:1/1:1/1:1/1	0/0:1/0:1/1:1/1	0/0:1/0:1/1:1/1	0/0:1/1:1/1:1/1	x/x:1/1:1/1:1/1	0/0:1/1:1/1:1/1	0/0:1/1:x/x:1/1	0/0:1/1:1/1:1/1
	0/0:1/1:1/1:1/1	0/0:1/1:X/X:1/1	0/0:1/1:1/1:1/1	0/0:1/1:1/1:1/1	0/0:1/1:1/1:1/1	1/0:1/1:1/1:1/1	0/0:1/1:1/1:1/2	0/0:1/1:1/1:x/x	0/0:1/1:x/x:x/x	0/0:1/1:1/1:1/1

**Figure 7.**
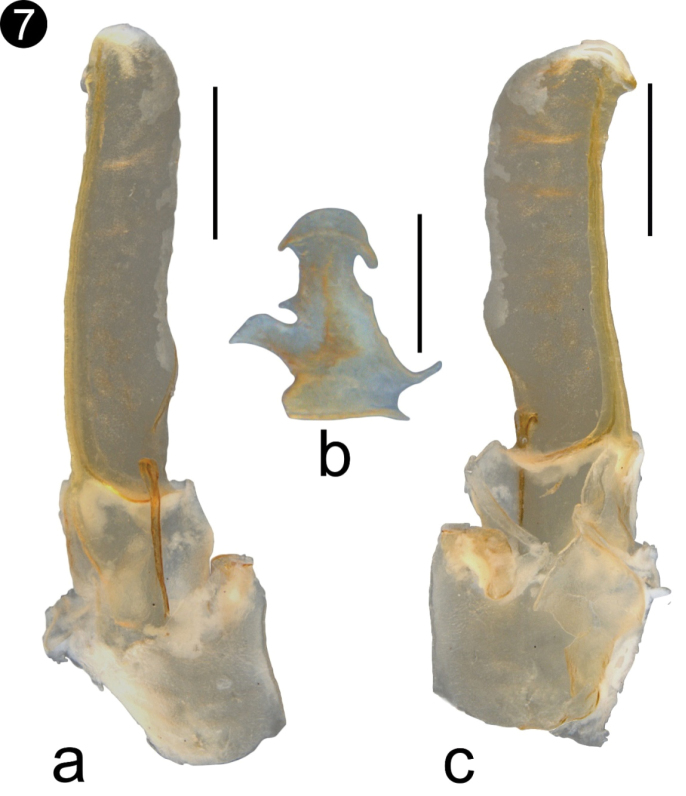
**a** Hemispermatophore of a paratype male of *Vaejovis islaserrano* sp. n. ectal view **b** Hemispermatophore of a paratype male of *V. islaserrano* sp. n. ental view. Scale bars: 0.5 mm **a, c**; 0.2 mm **b**.

*Hemispermatophore* (Fig. 7): Lamelliform (total length: 1.7; Lamella length: 1; width: 0.6mm). Lamella with a weak basal constriction at level of laminar hooks; dorsal trough long; mating plug present, with the distal barb margin smooth.

*Variation*: The sexual dimorphism in the species is little, but the total length of adult males and females differ by 18.3 to 20.3 mm on males and 20.3 to 24.1 mm on females; the presence of a white patch on mesosomal sternite V and the dorsal face of vesicle present on males and absent in females. The inner denticles, on the pedipalp chela movable finger, vary from five (on three specimens) to six (eight specimens). Carapace longer than pedipalp femur in males (CL/FL: 1.18) than in females (CL/FL: 1.5), but shorter than metasomal segment V (CL/MS V: 0.8) in males, whereas in females it is longer than metasomal segment V (CL/MS V: 1.33). Mesosomal sternite VII, setal counts ranges between eleven and twelve setae. Full variation of measurements is given in Table 1.

###### Distribution.

**Figure 8.**
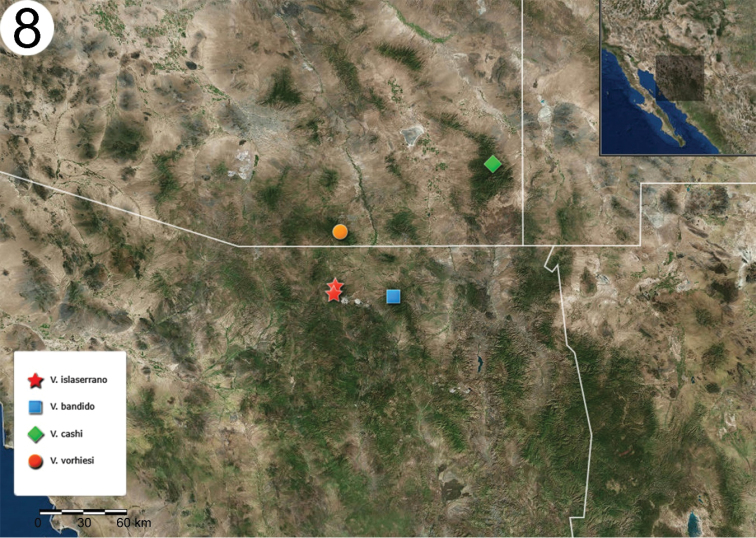
Map showing the type locality where *Vaejovis islaserrano* sp. n. was collected, and the distribution of the other three geographical and morphological closer species. Key: red star: *Vaejovis islaserrano* sp. n.; blue square: *V. bandido*; green rhombus: *V. cashi*; orange circle *V. vorhiesi*.

This species is known from a few localities in the higher elevations of the Sierra La Mariquita and Sierra La Elenita in Sonora, Mexico at 1911–2422 m. This currently represents the southwestern-most record for the “*vorhiesi*” group of the genus *Vaejovis* (Fig. 8).

###### Natural history

**Figure 9.**
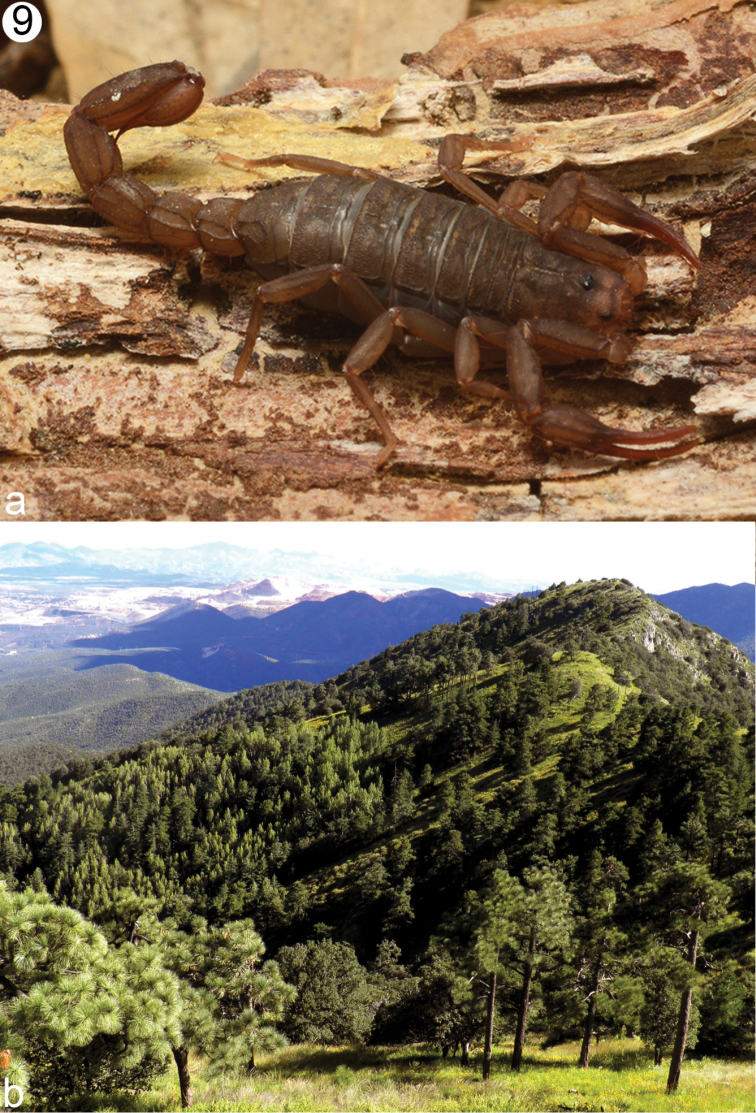
**a** Life female dorsal habitus in sight **b** type locality of *V. islaserrano* sp. n., showing the mixed pine-oak vegetation where it lives.

(Fig. 9). The specimens of *V. islaserrano* sp. n., were collected in August 2013 and September 2016. This species inhabits rocky slopes in pine-oak forest. (Fig. 9b). It was observed active on a cold rainy night, foraging in pine needle litter and living sympatric with *Paravaejovis spinigerus* (Wood, 1863), which inhabits open, rocky outcrops in the same areas.

## Supplementary Material

XML Treatment for Vaejovis islaserrano
